# AI-Assisted Spatial Metabolic Engineering in Plants: Integrating Flux Design, Spatial Omics, and Synthetic Biology

**DOI:** 10.3390/metabo16080519

**Published:** 2026-07-23

**Authors:** Huize Chen, Jia Yang, Meiting Du

**Affiliations:** 1 Shanxi Key Laboratory of Plant Macromolecules Stress Response, Taiyuan 030000, China; chenhz@sxnu.edu.cn (H.C.);; 2 School of Life Sciences, Shanxi Normal University, Taiyuan 030000, China

**Keywords:** plant metabolic engineering, spatial metabolomics, metabolic flux, plant synthetic biology, machine learning, plant biomanufacturing

## Abstract

**Background:** Plant synthetic biology reprograms metabolic networks for the sustainable production of high-value compounds. Recent computational advances incorporate machine learning to accelerate the design-build-test-learn (DBTL) cycle, enabling more predictable and scalable engineering in photoautotrophic chassis. However, the translation of AI-generated designs into stable plant phenotypes remains constrained by incomplete plant-specific training datasets, tissue heterogeneity, and limited in vivo validation. **Scope:** This review examines the convergence of machine learning methods with plant metabolic engineering across four spatial engineering levels: subcellular compartmentalization, cell/tissue/organ-specific control, developmental or inducible regulation, and genome-level organization. Spatial omics is considered a cross-cutting validation layer, and the evidence supporting each technology is classified as plant-demonstrated, non-plant proof-of-concept, or prospective. **Conclusions:** Integrating predictive machine learning with spatial engineering offers promising strategies to design complex biosynthetic pathways. Hybrid approaches, combining constraint-based metabolic models with generative algorithms, reduce trial-and-error in crop engineering. Future plant synthetic biology is likely to rely increasingly on automated and data-rich workflows to support more predictable plant bioproduction.

## 1. Introduction: Plant Synthetic Biology in the Algorithmic Era

Plant synthetic biology applies engineering principles to systematically reprogram plant metabolic pathways [1]. Plant chassis circumvent the post-translational glycosylation bottlenecks and low solubility limits commonly encountered in prokaryotic hosts by coupling photosynthetic carbon fixation with highly compartmentalized subcellular environments, enabling the synthesis of structurally complex natural products that are otherwise challenging to produce heterologously [2]. In this review, spatial metabolic engineering refers to the deliberate redistribution and coordinated control of enzymes, transporters, cofactors, intermediates, and storage processes across multiple spatial scales in plants. These spatial engineering levels comprise (i) subcellular compartmentalization and inter-organelle routing; (ii) cell-, tissue-, and organ-specific pathway expression; (iii) developmental-stage-specific or externally inducible regulation; and (iv) genome-level organization of multigene pathways. Spatial omics is treated as a cross-cutting measurement and validation layer rather than as an independent engineering scale. AI is considered a prioritization and data-integration layer operating across these levels rather than a substitute for plant experimentation.

To separate established evidence from future potential, the revised review uses three evidence categories: plant-demonstrated approaches supported by in planta metabolite, protein, or phenotype measurements; non-plant proof-of-concept approaches demonstrated in vitro, in microorganisms, or in animal cells; and prospective approaches whose application to plant metabolic engineering remains hypothetical or computationally inferred. Historically, research in this field focused primarily on optimizing the physical assembly of genetic circuits. While standardized DNA assembly frameworks, such as Golden Gate-based Modular Cloning (MoClo), have streamlined the physical assembly phase [3,4], they do not address the fundamental bottleneck located within the design phase. The multivariable nature of multi-gene expression dynamics within plant regulatory networks often eludes human intuition, where unpredictable epistatic interactions and metabolic burdens frequently arise. Machine learning algorithms address this regulatory multi-dimensionality, shifting the design-build-test-learn (DBTL) cycle from empirical screening toward a highly predictive, quantitative framework [5]. Deep learning models trained on specific multi-omics datasets computationally pre-screen circuit architectures, partially relieving the trial-and-error burden associated with multi-gene pathway construction [6].

Genomic language models have emerged as generative design engines. For instance, the Evo 2 model, a biological foundation model trained on 9 trillion DNA base pairs (the OpenGenome2 dataset) spanning all domains of life, processes a context window of 1 million tokens at single-nucleotide resolution [7]. This architecture can predict the functional impacts of genetic variation—from noncoding mutations to clinically relevant variants—without requiring task-specific fine-tuning.

However, while generalist biological foundation models like Evo 2 offer massive context windows and cross-domain zero-shot capabilities, they are trained predominantly on microbial and mammalian genomic datasets. Consequently, they often fail to fully decode the unique regulatory grammar of plants, which is characterized by highly repetitive heterochromatic regions, polyploid genomic complexities, and specific plant-specific cis-regulatory systems [8]. Nevertheless, the predictive capacity of such generalist genomic foundation models should not be directly equated with plant engineering performance. Plant genomes contain lineage-specific cis-regulatory elements, frequent whole-genome duplications, extensive repetitive sequences, and chromatin-dependent regulatory features that are underrepresented in many general biological training datasets. To address this limitation, the field has begun to move toward plant-specific genomic foundation models. A representative model is PlantGFM, a plant-oriented genomic foundation model built upon the Hyena operator architecture and pre-trained on 10.84 billion nucleotides from 12 representative plant species. Supporting a 64 kb context window, PlantGFM is optimized for the structural features of plant genomes and may enable the de novo design of novel plant-compatible genes. In initial validation trials, synthetic candidate sequences generated by PlantGFM successfully demonstrated transcription and translation in *Nicotiana benthamiana* leaves, suggesting the functional expression of generative plant DNA sequences. Importantly, detectable transcription or translation of synthetic sequences does not necessarily demonstrate predictable pathway-level function, stable inheritance, or agronomic utility. Therefore, plant-specific foundation models still require systematic validation across species, tissues, developmental stages, and environmental conditions.

Integrating this genomic foundation model with automated microfluidic screening provides a basis for a closed-loop platform. These automated infrastructures attempt to close the loop between algorithmic design and in vivo phenotypic validation, accelerating the transition of plant biomanufacturing toward a predictable, data-driven engineering science.

**Figure 1:**
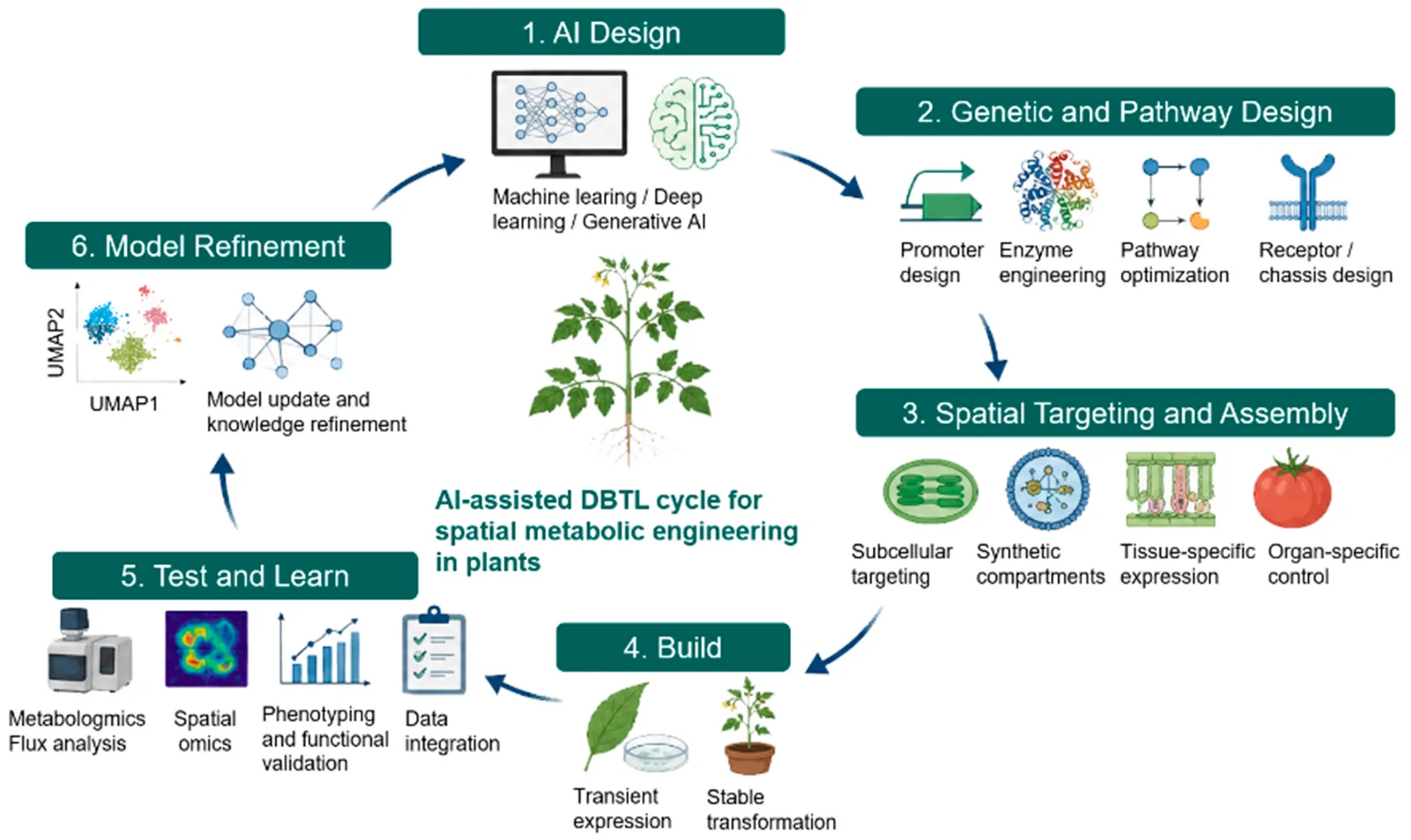
Conceptual framework of the AI-assisted design-build-test-learn cycle for plant spatial metabolic engineering. Machine learning and generative AI support metabolic flux prediction, pathway design, spatial targeting, plant-based construction, spatial omics-guided validation, and iterative model refinement.

The overall workflow proposed in this review is illustrated in Figure 1. AI-assisted design begins with computational prediction of genes, promoters, enzymes, and pathway architectures, followed by spatial targeting, plant-based construction, multi-omics validation, and iterative model refinement.

By emphasizing metabolic flux prediction, pathway compartmentalization, and spatial metabolomics-guided validation, this review highlights how AI-assisted design can be connected to measurable metabolic outputs in plant systems.

**Figure 2:**
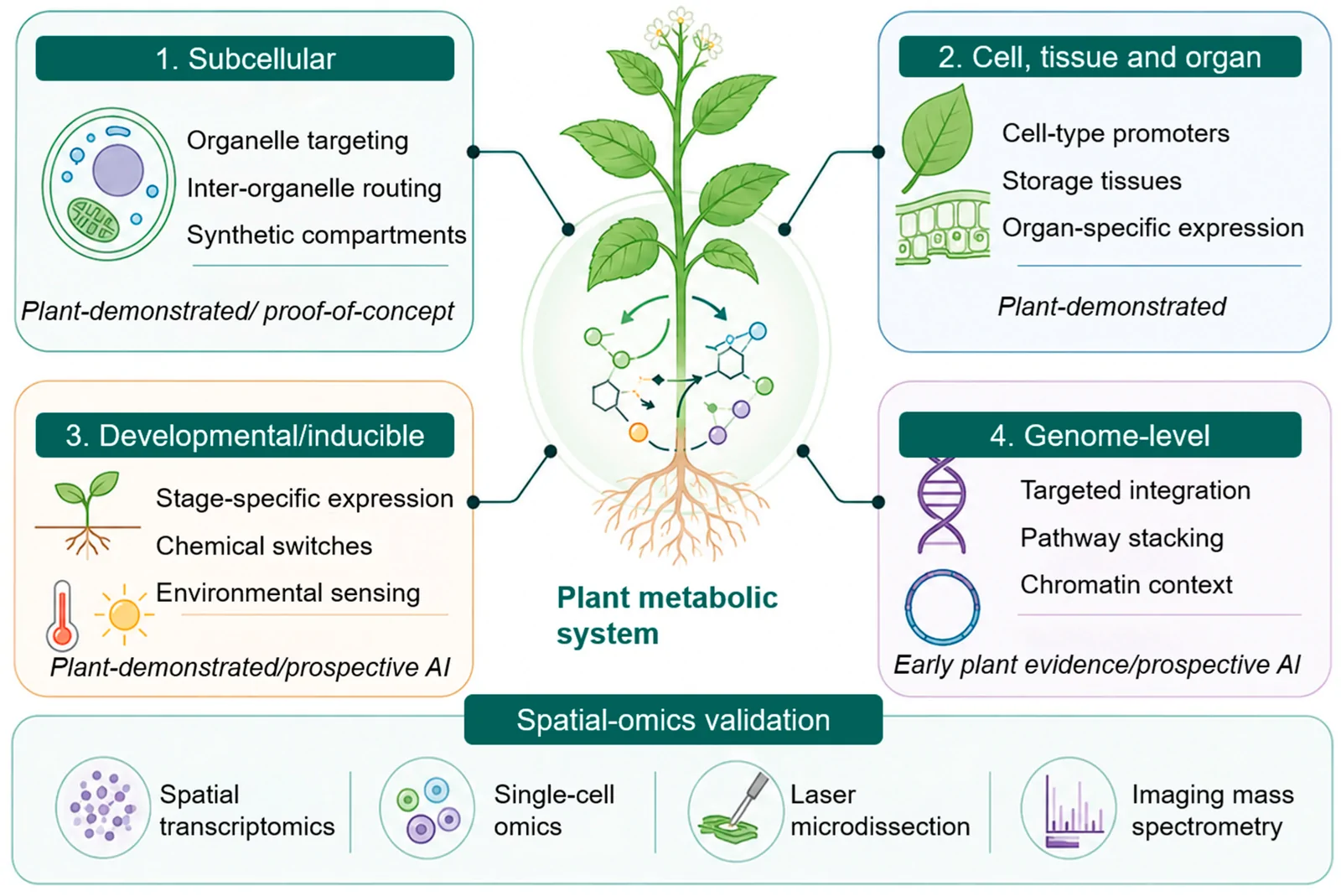
Spatial hierarchy and evidence levels of plant metabolic engineering. Spatial control operates at subcellular, cell/tissue/organ, developmental or inducible, and genome levels. Spatial transcriptomics, single-cell omics, laser microdissection, and imaging mass spectrometry form a cross-cutting validation layer that links computational prioritization to measured flux, product localization, transport, storage, and toxicity.

To make these spatial levels explicit, Figure 2 classifies the strategies reviewed below and places spatial transcriptomics, single-cell omics, laser microdissection, and imaging mass spectrometry as a cross-cutting validation layer.

## 2. Predictive Flux Optimization: From Metabolic Network Modelling to Pathway Balancing

The Push-Pull-Block (PPB) framework provides a practical logic for redirecting metabolic flux toward desired products. In this framework, precursor supply is enhanced through “push” strategies, product-forming reactions are strengthened through “pull” strategies, and competing endogenous sinks are reduced through “block” strategies [9]. In plant chassis, however, the effective implementation of PPB strategies requires more than the selection of a few pathway genes. It depends on the accurate identification of rate-limiting reactions, competing branches, transport steps, cofactor constraints, and tissue- or organelle-specific metabolic bottlenecks.

Constraint-based modelling has long provided a useful computational basis for metabolic flux analysis. Flux Balance Analysis (FBA), for example, defines a feasible flux space by requiring mass balance under reaction-capacity constraints, commonly represented as S·v = 0, where S is the stoichiometric matrix and v is the vector of reaction fluxes. Parsimonious FBA further narrows this solution space by assuming that cells tend to avoid unnecessary flux or enzyme investment. Although these methods are valuable for identifying potential intervention points, they remain limited by steady-state assumptions and incomplete metabolic annotations. These limitations are particularly important in plants, where metabolism is shaped by organellar compartmentation, diel regulation, cell-type specialization, source–sink transitions, developmental stage, and environmental responses. As a result, flux prediction pipelines developed in microbial or mammalian systems cannot be directly transferred to plant metabolic engineering without careful adaptation.

To overcome some of these limitations, recent systems biology approaches have increasingly integrated constraint-based modelling with supervised machine learning. Models such as Random Forest, XGBoost, and multilayer perceptrons can use transcriptomic, proteomic, metabolomic, and fluxomic datasets to predict metabolic fluxes and prioritize candidate engineering targets [10]. Compared with purely stoichiometric models, these data-driven approaches can better capture non-linear relationships between gene expression, enzyme abundance, and metabolic output. Nevertheless, unconstrained machine-learning models may generate predictions that are statistically accurate but biologically implausible, especially when they violate mass balance, thermodynamic feasibility, or known pathway architecture. Hybrid frameworks, including multi-omics-integrated neural networks and dynamic artificial metabolic networks, attempt to reduce this problem by embedding stoichiometric or mechanistic constraints into model training [11]. These approaches are conceptually important because they combine the flexibility of machine learning with the biological realism of genome-scale metabolic models.

Despite these advances, plant flux prediction remains far from fully reliable. Genome-scale metabolic models for plants are often incomplete, especially for specialized metabolism, transport reactions, and compartment-specific metabolite pools. Many secondary metabolic enzymes remain poorly annotated, and the same pathway may behave differently across species, organs, developmental stages, and environmental conditions. Therefore, computational flux predictions should be viewed as tools for candidate prioritization rather than definitive evidence of pathway performance. Experimental validation remains essential, particularly through isotope-labelling, targeted metabolomics, enzyme assays, and spatially resolved metabolite analysis.

Several recent examples illustrate how flux balancing can support plant-based production of high-value metabolites. In *Nicotiana benthamiana*, the citrus-derived flavone glycoside diosmin has been reconstructed through transient co-expression of a multi-gene flavonoid pathway, yielding 37.7 μg/g fresh weight, corresponding to approximately 61.95 nmol/g FW, as calculated from the reported mass-based yield [12,13]. This provides a useful quantitative benchmark for transient pathway reconstruction in leaves [14]. Similarly, multivariate modular optimization has been used to balance gene expression ratios within heterologous glucoraphanin biosynthetic modules, reducing intermediate accumulation and improving pathway performance [15,16]. These studies demonstrate that multi-gene pathway engineering in plants requires coordinated control of pathway dosage rather than simple overexpression of individual enzymes.

However, transient expression in *N. benthamiana* does not necessarily predict stable production in crops or long-term field performance. High-level expression of complex synthetic pathways can impose a metabolic burden, interfere with endogenous growth and defence programmes, or cause accumulation of toxic intermediates. Therefore, future pathway optimization will require dynamic and context-sensitive regulation rather than constitutive overexpression. AI-assisted promoter engineering provides one possible route toward this goal. For example, generative models trained on plant promoter datasets may help design synthetic promoters with tunable expression strength, enabling more precise control of pathway activation [17]. Such tools could be particularly useful for coordinating multi-enzyme pathways, reducing metabolic burden, and restricting expression to specific tissues or developmental stages.

At the same time, promoter activity measured in transient reporter assays should be interpreted cautiously [18]. Expression strength may change substantially in stable chromosomal contexts because of local chromatin state, DNA methylation, copy number variation, developmental regulation, and environmental responsiveness. Thus, AI-designed promoters require validation not only in transient assays but also in stable transgenic lines and, ultimately, under agronomically relevant conditions.

Accurate analytical feedback is also essential for closing the DBTL cycle. Targeted metabolite extraction, chemical derivatization, mass spectrometry, isotope tracing, and spatial metabolomics provide the experimental evidence needed to evaluate whether predicted flux redirection has actually occurred [19]. Without such feedback, computationally optimized designs may remain disconnected from plant physiological reality.

An evidence-gated validation sequence is therefore recommended: computational prediction and uncertainty ranking → transient plant expression → enzyme assays and isotope tracing → targeted metabolomics and spatial metabolite imaging → stable transformation and inheritance testing → field-relevant validation. Each step addresses a different failure mode. Transient assays test construct function and dosage; isotope tracing tests pathway flux; spatial measurements identify producing cells, transport routes, storage sites, and toxicity zones; stable lines reveal chromosomal and developmental effects; and field experiments quantify genotype-by-environment interactions. Computational output should therefore be treated as a ranked hypothesis until the relevant experimental gates have been passed [20,21,22].

Overall, machine learning can improve the identification of metabolic intervention points, but flux optimization alone is insufficient for plant synthetic biology. Once candidate pathways, enzymes, or promoters have been prioritized, their performance depends strongly on where they are placed within the plant cell or tissue. Enzyme localization, cofactor availability, organellar transport, intermediate diffusion, and tissue-specific expression all determine whether predicted flux improvements can be translated into functional biosynthesis. This spatial dimension provides the rationale for the subcellular and tissue-level engineering strategies discussed in the next section.

## 3. Architecting Subcellular Space: From Transit Peptides to Synthetic Organelles

### 3.1. Organelle-Targeting Peptides and Short Spatial Tags

At the subcellular level, spatial metabolic engineering involves the deliberate organization of enzymes and reactions across organelles, membranes, and synthetic compartments. Targeting entire heterologous metabolic cascades to specific subcellular compartments isolates reactions from cytosolic feedback inhibition, exploits localized cofactor pools, and mitigates intermediate toxicity. Subcellular localization predictors are therefore essential for the rational design of non-native transit peptides. DeepLoc 2.0, which leverages pre-trained protein language model (pLM) embeddings, achieves a state-of-the-art Matthews Correlation Coefficient (MCC) of 0.90 for predicting plastid-targeted proteins [23].

Advanced machine learning architectures, such as the attention-based neural networks developed [24], have demonstrated that deep learning can capture complex, non-linear sequence features to generate functional de novo signal peptides, surpassing traditional profile-based models in both targeting accuracy and sequence diversity. Mechanistic interpretability analyses of the attention layers in TargetP 2.0 and DeepLoc 2.0 revealed that the second residue following the initial methionine (position 2) exerts a dominant influence on chloroplast import classification [25]. Specifically, these neural networks identified that approximately two-thirds of chloroplast and thylakoid transit peptides possess an alanine at position 2, compared with only 20% in other endogenous plant proteins.

However, synthetic biologists must evaluate this structural feature with caution to avoid computational misinterpretations. In plant genetic engineering, an alanine codon (GCT or GCC) is frequently inserted at position 2 as an artificial cloning scar—often a direct consequence of engineering an NcoI restriction endonuclease site (CCATGG) to facilitate plasmid construction, or optimizing Kozak-like translation initiation contexts in plant expression cassettes [26]. Training biological language models on uncurated transgenic sequences containing these synthetic constructs introduces a systematic training bias, wherein the model misattributes high import-probability scores to an artificial translation enhancer rather than to a genuine evolutionary sorting signal [23]. Consequently, de novo transit peptide design must partition training data to filter out these non-natural sequence artifacts, ensuring that predictive algorithms capture only the genuine biophysical constraints of natural plastid import pathways. To circumvent these dataset-specific biases, recent computational efforts have shifted toward model architectures tailored specifically for photoautotrophic systems. A representative framework is Chlamy_ChloroPred, a deep learning-based binary classifier that couples ProtBERT-BFD protein language model embeddings with stacked bidirectional long short-term memory (BiLSTM) networks and an attentive pooling layer [24]. By extracting locality-aware features within the highly variable N-terminal ~50-amino-acid region of transit peptides, Chlamy_ChloroPred achieves a binary classification accuracy of 0.8462 for the *Chlamydomonas reinhardtii* proteome. Remarkably, despite being trained exclusively on microalgal datasets, Chlamy_ChloroPred exhibits effective cross-species transferability, predicting the chloroplastic proteome of the land plant *Arabidopsis thaliana* with an accuracy of 0.7316, representing a 12.6% improvement over the generalized TargetP 2.0. The integration of such interpretability-focused, lineage-specific networks offers a vital computational filter to distinguish genuine, evolutionarily conserved targeting signals from artificial cloning scars. Despite their utility, localization predictors mainly infer targeting probability rather than import efficiency, processing accuracy, enzyme folding, or catalytic competence after import. Therefore, predicted localization should be experimentally verified using fluorescent fusion proteins, immunoblotting of processed peptides, and subcellular metabolite profiling. Short peptides serve several distinct spatial functions and should not be treated as a single design class. Cleavable transit peptides deliver full-length enzymes to chloroplasts or mitochondria; signal peptides and retention motifs route proteins through the endoplasmic reticulum and Golgi; 15–20-residue encapsulation peptides recruit cargo to bacterial microcompartment shells; and designed interaction fragments may modulate protein assemblies or condensates. AI can rank candidate sequences by predicted targeting or interaction propensity, but cleavage efficiency, import kinetics, cargo stoichiometry, proteolytic stability, off-target localization, and catalytic competence must be measured experimentally.

### 3.2. Inter-Organelle Routing of Plant Metabolic Pathways

When engineered with precise biophysical constraints, compartmentalization within the plastidial stroma is a highly effective strategy for capturing the rich geranylgeranyl diphosphate (GGPP) precursor pool generated by the endogenous methylerythritol phosphate (MEP) pathway [15]. A prominent example is the metabolic reconstruction of early paclitaxel (Taxol) intermediates in *Nicotiana benthamiana*. By targeting the soluble Taxus-derived taxadiene synthase (TS) to the chloroplast stroma using a cleavable transit peptide, researchers achieved a taxadiene accumulation of 56.6 μg per gram of fresh leaf weight (μg/g FW). This example illustrates that spatial optimization is not simply a matter of placing all enzymes in the same compartment. Instead, successful engineering requires matching each enzyme with the membrane environment, redox partner, cofactor availability, and intermediate transport route required for catalytic function.

However, scaling this pathway to synthesize downstream oxidized taxanes introduces severe biophysical and spatial barriers. The subsequent oxidation step is catalyzed by taxadiene-5 α-hydroxylase (T5αH, a member of the CYP725A4 family), a hydrophobic cytochrome P450 monooxygenase that natively anchors to the endoplasmic reticulum (ER) membrane and requires an uninterrupted electron supply from its membrane-bound redox partner, NADPH-cytochrome P450 reductase (CPR). Native TS localizes to the chloroplast, whereas T5αH and CPR are associated with the endoplasmic reticulum, creating an initial spatial separation between the precursor-producing enzyme and the downstream P450 reaction. Co-expression of the enzymes in their native configurations did not produce detectable taxadiene-5α-ol, indicating that inter-organelle separation was a major pathway constraint. To overcome this limitation, Li et al. redirected a truncated T5αH–CPR fusion to the chloroplast using the transit peptide of TS, thereby co-localizing the committed biosynthetic reactions within the plastid. Further enhancement of the chloroplastic precursor pool through DXS and GGPPS expression increased taxadiene production to 56.6 ± 3.2 μg g^−1^ FW, while the optimized chloroplastic P450 configuration produced 1.3 ± 0.5 μg g^−1^ FW taxadiene-5α-ol [15].

Rather than solely relying on downstream pathway tuning to resolve this bottleneck, Edgar et al. demonstrated that rational engineering of TS itself can bypass downstream P450 limitations. By performing site-directed mutagenesis on the cyclase active site, they achieved a 2.4-fold improvement in yield and selectivity for an alternative cyclization product, taxa-4(20)-11(12)-diene, and for the Taxol precursor taxadien-5α-ol when coexpressed with CYP725A4, highlighting how upstream enzymatic optimization can alleviate downstream flux bottlenecks [15,27].

### 3.3. Synthetic Compartments and Encapsulation Peptides

Beyond exploiting endogenous lipid-bound compartments, an emerging direction in spatial metabolic engineering is the deployment of synthetic bacterial microcompartments (BMCs) as functional, proteinaceous organelles within plant cells [28]. BMC shell proteins and carboxysome components have been explored as modular scaffolds for plant synthetic biology, particularly in attempts to introduce carbon-concentrating compartments into tobacco chloroplasts. However, fully functional and broadly applicable synthetic BMC nanoreactors have not yet been established in plants [29]. In addition to plastidial targeting, the engineering of other organellar genomes, such as mitochondrial DNA base editing via mitoTALEN and mitoTALECD platforms, has emerged as a stable methodological route to reprogram organellar physiology, further expanding the cellular space available for compartmentalized metabolic engineering [30]. However, the deployment of BMCs in plant cells remains at an early proof-of-concept stage. Key unresolved issues include cargo loading efficiency, shell stability during plant development, possible interference with chloroplast physiology, metabolite permeability, and the energetic cost of producing large proteinaceous compartments.

These synthetic chloroplastic scaffolds can be functionalized by appending short (15 to 20 amino acids) encapsulation peptides (EPs) that fold into amphipathic α-helices to target cargo enzymes to the BMC lumen. Encapsulation of IspG and IspH may facilitate enzyme colocalization and locally organize terminal MEP-pathway reactions, although shell permeability to charged substrates, products, and cofactors remains a major unresolved constraint. Quantitative in vitro and computational molecular permeability measurements on *Haliangium ochraceum* BMC shells by researchers suggested that while the uncapped shell does not present an impenetrable barrier to small metabolic intermediates, it functions as a semi-permeable scaffold that can be biochemically tuned to partition targeted enzymes and substrates, providing critical biophysical validation for synthetic organelle designs [31]. More precisely, stopped-flow spectrophotometric assays evaluating the transport kinetics of small molecules across a 40 nm icosahedral *Haliangium ochraceum* BMC shell revealed that the intact, capped (fully assembled) shell imposes a distinct diffusion barrier, slowing downstream substrate–product exchange compared to free enzymes. Biophysical modeling of these transport kinetics demonstrated that capped shells are approximately 50-fold less permeable to substrates than their uncapped (vacant-vertex) counterparts [32]. This 50-fold permeability differential highlights a critical design trade-off in spatial metabolic engineering: while a fully sealed protein shell effectively sequesters volatile, toxic, or gaseous intermediates (such as isoprenoid precursors), it simultaneously restricts the passive influx of bulky hydrophilic cofactors like ATP and NADP. Consequently, the rational design of chloroplastic nanofactories must balance shell tiling stoichiometry and vertex capping efficiency to optimize pathway flux without starving encapsulated enzymes of essential energetic cofactors.

### 3.4. Spatial-Omics-Guided Mapping and Validation

To validate the efficacy of these subcellular interventions and confirm the tight confinement of metabolic pathways, spatial metabolomics techniques are important. Mass spectral imaging (MSI) provides label-free, high-resolution mapping of localized metabolites across single-cell boundaries and tissue layers [33]. By overlaying spatial metabolite profiles onto tissue anatomy, MSI allows researchers to assess whether engineered pathways alter local metabolite distribution and reduce interference with native cytosolic metabolism, confirming the physical containment of target products within engineered subcellular domains [33].

A major missing layer in current AI-assisted spatial metabolic engineering is spatially resolved biological data. Most computational models are trained on bulk transcriptomic, proteomic, or metabolomic datasets, which average signals across heterogeneous tissues and obscure cell-type-specific metabolic states. Spatial transcriptomics, single-cell multi-omics, imaging mass spectrometry, and spatial metabolomics can provide the anatomical and cellular context required to train plant-specific predictive models. These methods are particularly important for engineering pathways in tissues with strong metabolic zonation, such as leaves, roots, seeds, glandular trichomes, vascular tissues, and developing fruits.

Nevertheless, spatial omics remains technically challenging in plants because of cell walls, high vacuolar content, strong autofluorescence, specialized metabolites, and difficulties in preserving labile compounds during tissue sectioning. Therefore, future AI-guided spatial engineering should integrate bulk omics, single-cell data, spatial metabolite imaging, and targeted biochemical validation rather than relying on a single data modality.

The analytical modalities provide complementary information. Spatial transcriptomics can identify tissue domains in which pathway genes and transporters are co-expressed, whereas single-cell or single-nucleus RNA sequencing can resolve rare biosynthetic cell types and developmental trajectories [21,22]. Laser-capture microdissection followed by targeted metabolomics links defined anatomical regions to metabolite abundance and is particularly useful for vascular bundles, glandular tissues, seed coats, or storage parenchyma [20]. Imaging mass spectrometry can then map products and intermediates without relying on reporter genes. Integrating these datasets allows AI models to infer candidate bio-synthetic sites, source–sink transport routes, storage tissues, and zones in which toxic intermediates accumulate; these inferences still require targeted biochemical validation. Representative plant-based case studies and their spatial engineering outcomes are summarized in Table 1.

These cases show that spatial engineering can act through organelle targeting, inter-organelle partitioning, stable plastid–nuclear pathway distribution, and organ-specific pathway activation. The comparison also highlights why yield alone is insufficient: spatial localization, stability, transport, toxicity, inheritance, and environmental robustness must be evaluated together.

## 4. Secretory Pathway Trafficking and Glycoengineering

Spatial control in plant synthetic biology is not limited to small-molecule biosynthesis; it is also central to molecular farming, where recombinant protein folding, glycosylation, and accumulation depend on trafficking through the plant secretory pathway [36]. Transient expression using optimized viral vectors represents the current commercial standard [37]. The pEAQ-HT system, leveraging elements from the Cowpea Mosaic Virus and the P19 silencing suppressor, uncouples target mRNA translation from viral replication. This architecture drives expression limits up to nearly 50% of total soluble protein in leaves. Similarly, deconstructed viral vector systems like MagnICON exhibit high-yield metrics; for instance, the expression of the hepatitis B virus core antigen (HBcAg) achieved yields of 2.4 mg/g of fresh leaf biomass, assembling into highly immunogenic virus-like particles (VLPs) [38]. However, high transient expression does not necessarily ensure correct folding, homogeneous glycosylation, or scalable downstream purification. For pharmaceutical proteins, product quality, batch consistency, host–cell protein contamination, and regulatory comparability remain as important as expression yield.

Accordingly, this section addresses compartment-specific synthesis and processing of recombinant proteins rather than direct control of small-molecule flux. The relevant spatial variables are ER entry, Golgi transit, retention or secretion, cell-type expression, and the localization of host glycosyltransferase activity.

The viability of molecular farming to address global health emergencies was distinctly validated by the regulatory progression of Medicago’s Covifenz vaccine [39]. The regulatory trajectory of plant-made vaccines demonstrates the feasibility of the platform, but it also highlights that commercial success depends on manufacturing scale, cost competitiveness, public acceptance, and regulatory continuity. However, the immunogenicity of plant-specific N-glycans (specifically α-1,3-fucose and β-1,2-xylose linkages) remains a primary barrier to human therapeutics [40]. To humanize these glycan profiles, multiplex CRISPR/Cas9 editing has been employed to knock out glycosyltransferase genes in *Nicotiana benthamiana*. Researchers simultaneously targeted all seven glycosyltransferase homologs—including five α-1,3-fucosyltransferase genes and two β-1,2-xylosyltransferase genes (specifically targeting the previously omitted FucT5)—using multiplex CRISPR/Cas9 editing to generate stable, Cas9-free, homozygous glycoengineered *Nicotiana benthamiana* lines [41]. A remaining challenge is that humanized N-glycosylation is protein-dependent. Removal of plant-specific glycans reduces immunogenic risk, but it does not automatically generate fully human-like glycoforms for every recombinant protein. Given the high polyploid complexity and functional redundancy within the *Nicotiana benthamiana* genome, targeting all seven glycosyltransferase loci is necessary to minimize residual activity, as the previously neglected *FucT5* homolog can exert residual or conditional α-1,3-fucosyltransferase activity. Recent multiplex CRISPR/Cas9 editing pipelines utilizing non-repetitive guide RNA arrays successfully achieved complete, simultaneous knockouts of all seven glycosyltransferase loci in the T0 generation (specifically lines HL40 and HL64), carrying frame-shift indels up to 26 bp [41]. Subsequent segregation screening in the T1 and T2 generations isolated stable, homozygous lines that were fully Cas9-free. Removing the Cas9 transgene not only stabilizes the edited loci against further nuclease activity and off-target mobilization, but also aligns the resulting molecular farming platform with international biosafety standards, significantly lowering regulatory barriers and easing public acceptance for plant-produced human therapeutics. This strategy can be viewed as engineering of secretory-pathway processing, because glycan maturation is progressively shaped as proteins move from the ER to the Golgi apparatus [42]. The resulting engineered lines produce recombinant proteins with N-glycans lacking the major plant-specific β1,2-xylose and core α1,3-fucose residues.

The editing challenge in *N. benthamiana* is amplified by its polyploid-derived and highly duplicated genome. Extensive homeologous gene retention means that several functionally redundant copies can contribute to the same glycosylation reaction; disrupting only one or a subset of homologues may therefore leave residual activity [43]. Multiplex editing must also distinguish genuine paralogues from assembly artefacts and should be followed by segregation, off-target assessment, glycan profiling, and evaluation of growth and fertility.

## 5. Cellular and Tissue-Level Spatial Dynamics: AI-Guided Receptor Design

Reprogramming metabolic and water flux across plant tissues also demands spatial control at cellular and stomatal levels. Engineering crops for climate resilience requires interventions that do not inherently suppress vegetative growth or biomass yield. Synthetic immunity provides a framework to decouple stress tolerance from growth-yield penalties by deploying programmable genetic circuits and orthogonal receptor systems [1].

An early example of this cellular-level spatial control is the development of PYR1-Mandi, a modified abscisic acid receptor with nanomolar sensitivity to the fungicide mandipropamid [44,45]. During water deficits, plants natively produce elevated levels of ABA to control guard cell aperture and promote drought tolerance. Utilizing structure-guided rational design and yeast-two-hybrid screening, researchers mutated six residues within the PYR1 ligand-binding pocket to shift its sensitivity from ABA to mandipropamid [46]. In transgenic plants, mandipropamid activated PYR1-Mandi-dependent ABA signalling, promoted stomatal closure, and reduced water loss [47]. This strategy is conceptually powerful, but field-level deployment would require careful evaluation of ligand persistence, off-target agrochemical effects, environmental safety, cost, and regulatory acceptance. Beyond its utility as a bespoke chemical switch, the PYR1-HAB1 chemically induced dimerization (CID) system is increasingly recognized as a highly programmable, generic scaffold for broad-spectrum small-molecule sensing due to its unique allosteric amplification mechanism [45]. While ligand binding occurs solely within the malleable pocket of the PYR1 monomer, subsequent recruitment of the PP2C coreceptor (such as HAB1 or ABI1) stabilizes the complex, lowering the ligand off-rate and boosting the apparent sensitivity of micromolar binders up to 100-fold in vivo. Capitalizing on this ‘antibody-like’ simplicity, recent high-throughput screens utilizing engineered PYR1 double-site mutant (DSM-Hao) and triple-site mutant (TSM) libraries against 2726 diverse ligands successfully isolated functional sensors for 181 structurally distinct molecules—including plant natural products, the explosive 2,4,6-trinitrotoluene (TNT), and per- and polyfluoroalkyl substances (PFAS)—achieving an overall success rate of 6.6% [47]. This provides a possible pipeline where fixed-conformer docking and rigid-body alignment algorithms can rapidly repurpose the START domain of PYR1 into a target-driven biosensing platform, bypassing the historical constraints of empirical trial-and-error mutagenesis. However, broad ligand responsiveness may also increase the risk of unintended activation by structurally related environmental compounds. Therefore, receptor engineering should balance sensitivity, specificity, reversibility, and ecological safety.

While PYR1-Mandi demonstrated the feasibility of chemical ligand-receptor hijacking via classic structural biology, AI-based screening may shorten the early stages of receptor engineering. Modern machine learning docking models, generative structural predictors, and deep learning-based activity engines can evaluate millions of virtual mutations in silico, calculating binding free energies for novel ligand–receptor pairs before physical synthesis starts [48]. This approach transitions receptor engineering from empirical mutagenesis to target-driven, computational design. When coupled with tissue-specific promoters or synthetic metabolic modules, such engineered receptors could provide inducible control over metabolic fluxes in defined cell types or organs.

For plant breeders, AI is most useful when it augments rather than replaces expert intuition. Breeders can use high-dimensional genomic, phenomic, environmental, and management data to rank crosses, identify non-obvious genotype-by-environment interactions, and quantify uncertainty, while retaining biological knowledge to reject implausible recommendations [49]. Further progress requires interpretable feature attribution, uncertainty calibration, transfer learning across germplasm and environments, standardized phenotyping, and prospective multi-location trials. For tissue-level control specifically, model predictions must also be validated for cell-type specificity, inducibility, reversibility, developmental stability, and unintended effects on growth and reproduction.

## 6. Genomic Circuitry: Artificial Chromosomes and Chromatin Topology

Beyond cellular signalling modules, scalable plant engineering also requires genome-level strategies that can maintain large synthetic pathways across generations. Scaling metabolic engineering requires the simultaneous transfer of entire multi-gene pathways, often exceeding 50 kb in size. Standard Agrobacterium-mediated integration results in random genomic insertions, leading to unpredictable positional effects and severe epigenetic silencing. Engineering synthetic plant chromosomes or utilizing targeted integration sites may provide a route for modular pathway stacking [50]. However, synthetic chromosome engineering in plants remains less mature than in yeast or mammalian systems. Stable maintenance, meiotic transmission, centromere function, epigenetic silencing, and transformation efficiency remain major barriers.

Plant artificial-chromosome technology is therefore relevant as a long-term pathway-stacking strategy, but it is not yet a routine engineering platform. Engineered maize minichromosomes have been generated by telomere-mediated truncation and by in vitro assembly, demonstrating that autonomous or truncated chromosome platforms are biologically possible [51,52,53]. Their broader use remains limited by low recovery frequency, rearrangement, variable meiotic transmission, centromere epigenetics, loading of large DNA cargoes, and species-dependent transformation. Engineered minichromosomes should not be confused with polytene chromosomes. Polytene chromosomes arise through repeated DNA replication without cell division in specialized cells and are not currently established as artificial vectors for transferring multigene pathways into crops.

Sequence-based models developed primarily in mammalian systems, including Akita [54] and Orca [50], illustrate how DNA sequence can be used to predict aspects of three-dimensional genome organization. Their application to plant chromatin and transgene landing-pad selection remains prospective and requires benchmarking with plant Hi-C and transgene-expression datasets. By predicting topologically associating domains (TADs) and boundary elements, these tools may help researchers prioritize candidate genomic landing pads—stable chromosomal loci insulated from the suppressive spread of heterochromatin. Because most 3D genome prediction models have been developed using animal or human datasets, their direct applicability to plant genomes requires careful benchmarking using plant Hi-C, epigenomic, and transgene-expression datasets. In principle, directing CRISPR-Cas9 to candidate landing pads could improve targeted integration and reduce positional effects, although stable inheritance and expression of large synthetic operons in plants remain to be systematically validated.

## 7. Generative AI: From Structural Prediction to De Novo Protein Design

The integration of specialized AI tools has transitioned from analyzing biological structures to explicitly generating them. The latest iteration of predictive models, AlphaFold 3, expands modeling parameters beyond single-chain proteins. By replacing previous architectures with the Pairformer module, it improves the modelling of protein–ligand, protein–nucleic acid, and multi-chain complexes [55,56]. This capacity to directly model molecular interactions reduces barriers in understanding enzyme-substrate transitions in complex plant metabolic nodes. Although AlphaFold 3 improves molecular interaction modelling, predicted binding poses or complex structures should not be interpreted as direct evidence of catalytic activity. Enzyme engineering still requires kinetic assays, substrate specificity tests, and validation under plant-relevant pH, redox, and cofactor conditions.

These models predict intermediate representations rather than plant performance. AlphaFold 3 can propose structures and interaction geometries, but it does not establish catalytic turnover, substrate specificity, product profile, compartment-specific folding, or metabolic flux. RFdiffusion [57] generates candidate backbones, and ProteinMPNN [58] proposes sequences compatible with a backbone, but neither guarantees expression, stability, cofactor use, or activity in a chloroplast, ER, vacuole, or cytosol. Promoter and genomic foundation models similarly estimate sequence–activity relationships within their training domain; they do not directly predict chromatin effects, stable inheritance, environmental responsiveness, or agronomic phenotype.

Protein design has expanded beyond structural prediction to de novo backbone generation [59]. While earlier iterations required predefined scaffold libraries, architectures like RFdiffusion can generate candidate protein backbones around predefined structural motifs or active-site geometries. Once the 3D backbone is formulated, graph neural networks like ProteinMPNN map the coordinates back into amino acid sequences optimized for heterologous expression. To complement de novo scaffold generation, the downstream optimization of complex multi-enzyme cascades requires fast, cooperative evolution. The recently developed MULTI-evolve framework addresses this by combining protein language models with epistatic modeling to predict synergistic multi-site mutations [60]. When paired with high-efficiency MULTI-assembly mutagenesis, this pipeline systematically engineers functional variants across multikilobase sequences, achieving up to 10-fold catalytic improvements in a single round of machine learning-guided evolution [57,58]. In plant metabolic engineering, de novo-designed enzymes face additional constraints, including chloroplast or ER folding environments, post-translational modification, codon usage, protein turnover, and possible toxicity of novel intermediates.

AI may provide new opportunities for physical control over the cellular space by regulating biomolecular condensates. The AI-driven FragFold method computationally designs protein fragments to control liquid–liquid phase separation (LLPS). FragFold-like strategies may provide a conceptual basis for future plant applications [61,62,63]. Technologically, FragFold employs a computational pipeline to screen isolated peptide fragments against their full-length parental proteins, identifying specific interfaces that can be targeted to modulate phase separation. This approach operates via two distinct biophysical mechanisms: monovalent inhibition, where designed fragments competitively block the weak, multivalent domain–domain interactions (such as the FERM-kinase domain interface) required for phase separation; and bridging enhancement, where fragments act as non-covalent cross-linkers that physically strengthen the macromolecular network. In experimental validations, FragFold-designed fragments achieved a 50% success rate (9/18 designs) in controlling condensate dynamics across structurally diverse proteins (including G3BP1 and TDP-43) both in vitro and in living cells [63]. Deploying these short peptide modulators provides an alternative to overexpressing bulky, endogenous intrinsically disordered regions, as it may reduce the burden associated with overexpressing large intrinsically disordered regions. The spatially targeted delivery of these engineered condensates into chloroplasts may provide a future strategy for membrane-free spatial organization of metabolic enzymes. In plant metabolic engineering, designed peptide fragments could, in principle, be used to organize artificial membrane-free condensates within the chloroplast stroma or cytosol, physically sequestering volatile or toxic secondary metabolites without constructing physical lipid membrane barriers. Because current evidence for FragFold-like condensate control is largely derived from non-plant systems and includes preprint-level findings, its application to plant metabolic engineering should be presented as a future possibility rather than an established platform.

Plant cells already use biomolecular condensates in signalling, development, RNA regulation, and stress responses [64], but this biological precedent does not yet demonstrate that designed peptide fragments can increase plant metabolic flux. FragFold is cited as a non-plant proof-of-concept and preprint-level study. Plant application would require demonstration of controllable assembly and dissolution, enzyme partitioning, metabolite permeability, material-state measurements, organelle compatibility, and a measurable improvement in pathway output without toxicity or growth penalties.

A more detailed tool-level comparison of representative AI-assisted technologies, their applications, advantages, and limitations is provided in Supplementary Table S1. To distinguish established plant applications from emerging or speculative technologies, Table 2 summarizes the major technology layers, their current evidence status, and the principal barriers to implementation in plant spatial metabolic engineering.

## 8. Conclusions and Future Perspectives

Plant biomanufacturing increasingly depends on the integration of precise genetic perturbation, spatial pathway organization, and AI-assisted design. The maturation of the Push-Pull-Block paradigm, coupled with advancements in synthetic compartmentalization and viral vector-driven molecular farming, provides useful platforms for generating high-value biomolecules. Despite these advances, several bottlenecks remain unresolved. First, plant-specific AI models are limited by the scarcity of standardized, high-quality training datasets. Second, many computational predictions are validated only in transient expression systems rather than stable plant lines. Third, the spatial organization of metabolism is influenced by development, environment, organellar physiology, and tissue context, which are difficult to capture using bulk omics datasets. Finally, regulatory approval, field performance, biosafety, and public acceptance will determine whether AI-designed plant systems can move beyond laboratory proof-of-concept studies.

Achieving a more predictive design-build-test-learn cycle remains constrained by data availability. Current generative AI models and protein language models face a major limitation: many are trained predominantly on microbial and mammalian datasets. They may incompletely capture the unique biophysical constraints of plant microenvironments, such as the specific pH gradients or redox potentials within chloroplasts and vacuoles. Overcoming this requires the establishment of automated plant biofoundries. Therefore, the future success of AI-assisted plant engineering will depend not only on the generation of candidate genetic designs, but also on metabolite-level validation through flux analysis, targeted metabolomics, and spatially resolved metabolic profiling. High-throughput, standardized data collection from single-cell plant omics is essential to fine-tune existing language models. Once the loop between in silico generation and high-throughput in vivo plant validation is closed, plant synthetic biology may gradually move from empirical optimization toward more predictable and data-supported engineering.

AI should therefore be framed as a prioritization and hypothesis-generation tool, not as a replacement for experimental validation or breeder expertise. Plant metabolic phenotypes emerge from genetic background, cell identity, developmental stage, organellar physiology, transport, environment, and management. The most credible future workflows will report model uncertainty, distinguish interpolation from extrapolation, and require metabolite-level, spatial, stable-line, inheritance, and field validation before claiming improved pathway performance.

## Figures and Tables

**Table 1:** Plant-based case studies illustrating spatial strategies, metabolic outcomes, and unresolved bottlenecks.

Plant Chassis	Target Pathway/Product	Spatial Strategy	Reported Outcome	Remaining Bottleneck
*N. benthamiana*	Diosmin/flavonoid pathway	Transient reconstitution of ten diosmin-biosynthetic genes organized into three multigene modules in leaves	37.7 μg/g FW diosmin [12]	Transient output does not establish stable inheritance, tissue portability, or field performance
*N. benthamiana*	Taxadiene and taxadien-5α-ol	Chloroplast targeting of TS and a transit-peptide-directed truncated T5αH–CPR fusion, combined with DXS/GGPPS-mediated precursor enhancement	56.6 ± 3.2 μg/g FW taxadiene and 1.3 ± 0.5 μg/g FW taxadien-5α-ol [15]	Inter-organelle transfer, P450 redox coupling, and downstream oxidation remain limiting
*N. tabacum*	Artemisinic-acid pathway	Combinatorial nuclear supertransformation of a transplastomic recipient line (COSTREL)	120.4 ± 42 mg/kg FW artemisinic acid in the highest-performing T1 line, representing an increase of up to 77-fold relative to the recipient line [34]	Large genotype-to-genotype variation and the need to balance plastid and nuclear expression
*S. lycopersicum*	Anthocyanin biosynthesis	Fruit-specific expression of the snapdragon transcription factors Del and Ros1	Anthocyanin concentrations comparable to those of blackberries and blueberries, accompanied by an approximately threefold increase in hydrophilic antioxidant capacity [35]	Developmental promoter dependence, metabolic burden, and stability across cultivars and environments

Note: Reported outcomes reproduce quantitative or qualitative findings from the cited primary studies. The remaining bottlenecks represent the authors’ critical synthesis based on the experimental systems and limitations described in those studies.

**Table 2:** Major technology layers, metabolic applications, and current bottlenecks in AI-assisted spatial metabolic engineering in plants.

Technology Layer	Representative Tools or Strategies	Main Applications in Plant Synthetic Biology	Current Bottlenecks and Unresolved Issues	Evidence Status
Sequence-level AI design	Genomic foundation models, promoter design models, regulatory sequence predictors	Gene discovery, synthetic promoter design, regulatory element optimization, variant-effect prediction	Limited plant-specific training datasets, weak interpretability, insufficient validation across species, tissues, developmental stages, and environmental conditions	Early plant proof-of-concept for plant-specific genomic models; most generative applications remain prospective
Flux and pathway optimization	FBA, pFBA, machine learning-based flux prediction, multi-omics-constrained metabolic models	Identification of rate-limiting reactions, pathway balancing, precursor supply optimization, reduction in competing metabolic sinks	Incomplete plant metabolic models, poorly annotated specialized metabolism, uncertain transport reactions, and limited compartment-specific flux data	Plant-demonstrated for metabolic modelling and pathway optimization, although many AI models are trained or benchmarked mainly in microbial and mammalian systems
Subcellular targeting and compartmentalization	Transit peptide design, protein localization predictors, chloroplast targeting, ER–plastid pathway partitioning	Organelle-specific pathway construction, improved precursor utilization, reduction in intermediate toxicity, cofactor-aware pathway design	Predicted localization does not guarantee import efficiency, correct processing, protein folding, catalytic activity, or compatibility with local redox and cofactor environments	Plant-demonstrated for chloroplast, mitochondrial, ER, Golgi, vacuolar, and tissue-specific targeting; AI-guided de novo targeting-sequence design remains early-stage
Synthetic organelles and spatial scaffolds	Bacterial microcompartments, encapsulation peptides, synthetic chloroplastic scaffolds, proteinaceous nanoreactors	Enzyme clustering, metabolite sequestration, pathway insulation, prevention of volatile or toxic intermediate loss	Cargo loading efficiency, shell permeability, developmental stability, energetic burden, organelle compatibility, and scalability remain poorly resolved	Early plant proof-of-concept for carboxysome-related and shell-protein components; broadly functional BMC nanoreactors remain prospective
Secretory pathway and glycoengineering	Viral expression vectors, ER/Golgi targeting, multiplex CRISPR/Cas9-mediated glycan remodeling	Recombinant protein production, vaccine manufacturing, therapeutic protein expression, humanized glycosylation	Product quality consistency, protein-specific glycoform variation, downstream purification, regulatory approval, and large-scale manufacturing remain major challenges	Plant-demonstrated and commercially relevant for transient expression and glycoengineering
Cellular, tissue, and organ-level control	Synthetic receptors, chemically inducible dimerization systems, tissue-specific promoters, AI-assisted receptor design	Environmental sensing, inducible pathway activation, stomatal regulation, stress-responsive metabolic or signalling control	Field-level ligand persistence, unintended receptor activation, ecological safety, specificity, reversibility, and regulatory acceptance require further evaluation	Plant-demonstrated for tissue-specific expression and PYR1-MANDI; broad AI-guided receptor redesign remains prospective
Genome-level pathway stacking	CRISPR-based targeted integration, landing-pad prediction, artificial chromosomes, chromatin-aware transgene design	Stable multigene pathway assembly, reduced positional effects, long-term inheritance of complex synthetic traits	Plant artificial chromosome systems remain immature; epigenetic silencing, meiotic stability, transformation efficiency, and reliable landing-pad prediction remain limiting	Early plant evidence for engineered minichromosomes and targeted integration; routine artificial-chromosome deployment remains prospective
Generative protein and spatial validation platforms	AlphaFold 3, RFdiffusion, ProteinMPNN, condensate engineering, spatial omics, automated DBTL platforms	Enzyme redesign, catalytic optimization, membrane-free compartmentalization, spatial validation, iterative model refinement	Most designs require extensive experimental validation; plant-specific folding environments, subcellular physiology, data standardization, and biofoundry infrastructure remain insufficient	Predominantly non-plant proof-of-concept; plant metabolic applications remain largely prospective
Biomolecular condensate engineering	FragFold-like peptide design, phase-separation modulators, synthetic interaction fragments	Membrane-free enzyme organization, dynamic pathway clustering, and potential sequestration of selected enzymes or intermediates	Limited peer-reviewed validation, uncertain condensate stability in plant cells, possible interference with endogenous phase-separated systems, uncontrolled material properties, off-target interactions, and unknown physiological burden	Non-plant proof-of-concept, including preprint-level evidence; plant applications are prospective
Spatial-omics-guided validation	Spatial transcriptomics, single-cell or single-nucleus RNA sequencing, laser-capture microdissection, imaging mass spectrometry, spatial metabolomics	Identification of biosynthetic cell types, mapping of pathway expression, metabolite localization, transport-route inference, storage-site identification, and toxicity assessment	High cost, limited throughput, tissue-sectioning artefacts, cell-wall constraints, metabolite instability, autofluorescence, incomplete cross-platform standardization, and difficulties linking spatial abundance to actual metabolic flux	Plant-demonstrated for spatial mapping and cell-type resolution; integration with AI-guided metabolic engineering remains emerging
Integrated AI–DBTL workflow	High-throughput screening, robotics, microfluidics, multi-omics feedback, spatial metabolomics	Closed-loop design-build-test-learn optimization for predictable plant biomanufacturing	Infrastructure-intensive; plant systems remain slower and less standardized than microbial chassis; stable in planta validation is still a major bottleneck	Early-stage in plants; more mature in microbial systems

## Data Availability

No new data were created or analyzed in this study. Data sharing is not applicable to this article.

## Supplementary Materials

The following supporting information can be downloaded at: https://www.mdpi.com/article/10.3390/metabo16080519/s1, Table S1: Detailed tool-level comparison of AI-assisted technologies for plant spatial metabolic engineering.
