# Supplementary material for: AI-Assisted Spatial Metabolic Engineering in Plants: Integrating Flux Design, Spatial Omics, and Synthetic Biology
Source: Metabolites. 2026 Jul 23;16(8):519. doi: 10.3390/metabo16080519 (PMC13515040; doi:10.3390/metabo16080519)
Supplement: Supplementary file 1 [file metabolites-16-00519-s001.zip › metabolites-4425840-supplementary.pdf]

Supplementary

Table S1. Detailed tool-level comparison of AI-assisted technologies for plant spatial metabolic engineering.

Technology Category	Representative Tools/Approaches	Major Applications in Plant Synthetic Biology	Current Advantages	Major Limitations and Challenges
Genomic Foundation Models	Evo 2, PlantGFM, DNABERT, Nucleotide Transformer	Gene discovery, promoter engineering, regulatory sequence design, variant effect prediction	Enable large-scale sequence learning and generative design; accelerate candidate prioritization	Limited plant-specific training datasets; poor interpretability; insufficient validation across diverse plant species and environmental conditions
Machine Learning-Assisted Flux Modeling	FBA, pFBA, MINN, dAMN, XGBoost, Random Forest, Neural Networks	Metabolic flux prediction, pathway optimization, bottleneck identification	Integrates multi-omics information; improves prediction accuracy beyond traditional constraint-based models	Incomplete metabolic networks, uncertain transport reactions, limited compartment-specific data, and poor transferability between species
AI-Guided Promoter Engineering	TargetGAN, STARR-seq-assisted design platforms	Synthetic promoter design, dynamic pathway regulation, tissue-specific expression control	Enable programmable transcriptional regulation and de novo promoter generation	Long-term genomic stability, chromatin effects, epigenetic silencing, and field-level performance remain poorly understood
Protein Localization Prediction	DeepLoc 2.0, TargetP 2.0, Chlamy_ChloroPred	Transit peptide design, organelle targeting, subcellular pathway compartmentalization	Rapid identification of candidate targeting sequences; facilitates rational spatial engineering	Predicts localization probability rather than actual import efficiency, protein folding, or enzymatic activity
Spatial Metabolic Engineering	Chloroplast targeting, ER-plastid	Enhancement of precursor utilization, reduction in metabolic	Improves pathway efficiency and	Organellar transport constraints,

	partitioning, transit peptide engineering	interference, compartment-specific pathway optimization	minimizes intermediate toxicity	cofactor availability, redox compatibility, and metabolite exchange remain difficult to predict
Synthetic Organelles and Bacterial Microcompartments (BMCs)	Synthetic BMCs, encapsulation peptides, chloroplastic nanofactories	Metabolite sequestration, pathway insulation, prevention of volatile intermediate loss	Provides physical confinement of metabolic pathways and localized enzyme clustering	Cargo loading efficiency, shell permeability, developmental stability, energetic burden, and scalability remain unresolved
Molecular Farming and Glycoengineering	pEAQ-HT, MagnICON, multiplex CRISPR/Cas9 glycoengineering	Recombinant protein production, vaccine manufacturing, therapeutic protein expression	High expression yields and customizable glycosylation profiles	Product quality consistency, regulatory approval, downstream purification, and protein-specific glycosylation complexity
Synthetic Receptor Engineering	PYR1-Mandi, CID systems, AI-assisted receptor design	Environmental sensing, stress-responsive regulation, orthogonal signaling pathways	Enable programmable control of plant responses using external molecules	Specificity, ecological safety, field deployment, and unintended ligand recognition require further investigation
Genome Engineering and Artificial Chromosomes	CRISPR/Cas9, AI-assisted landing-pad prediction, synthetic chromosomes	Large-scale pathway stacking, stable multigene integration	Potential solution for complex pathway assembly and predictable transgene expression	Plant artificial chromosome technology remains immature; epigenetic silencing and chromosomal stability remain major barriers
Generative Protein Design	AlphaFold 3, RFdiffusion, ProteinMPNN, MULTI-evolve	Enzyme design, catalytic optimization, protein engineering	Expands accessible protein sequence space and accelerates enzyme development	Most designs require extensive experimental validation; plant-specific cellular

				constraints are often ignored
Biomolecular Condensate Engineering	FragFold, LLPS-based engineering	Membrane-free compartmentalization, dynamic metabolite sequestration	Provides a novel strategy for spatial organization without membrane construction	Evidence in plants is currently limited; most demonstrations remain in non-plant systems
Spatial Omics and Single-Cell Technologies	Spatial transcriptomics, spatial metabolomics, MSI, single-cell multi-omics	Cell-type-specific pathway analysis, AI model training, spatial validation of engineered systems	Generates spatially resolved biological information critical for precision engineering	High cost, technical complexity, limited throughput, and challenges associated with plant tissue processing
Automated Biofoundries and DBTL Platforms	High-throughput screening, robotics, microfluidics, AI-assisted DBTL pipelines	Closed-loop design-build-test-learn cycles	Accelerates iterative optimization and reduces experimental workload	Infrastructure-intensive; standardized datasets and plant-specific automation platforms remain scarce
